# Melatonin as a Smart Protector of Pregnancy in Dairy Cows

**DOI:** 10.3390/antiox11020292

**Published:** 2022-01-31

**Authors:** Essa Dirandeh, Zarbakht Ansari-Pirsaraei, William Thatcher

**Affiliations:** 1 Department of Animal Science, Sari Agricultural Sciences and Natural Resources University, Sari, Mazandaran 578, Iran; ansari2000@yahoo.com; 2 Department of Animal Sciences, University of Florida, Gainesville, FL 32611, USA; thatcher@ufl.edu

**Keywords:** melatonin, embryonic loss, redox status, TBARS, AOPP

## Abstract

The experimental objective was to examine the role of melatonin and its pathways in the maintenance of pregnancy in lactating dairy cows. Blood samples were collected at days 0, 16 and 32 after timed AI from cows (n = 200) in order to consider plasma melatonin concentrations and to conduct AOPP (advanced oxidation products of proteins) and TBARS (thiobarbituric acid reactive substances) tests. Luminal endometrial cells were collected at day 16 using a Cytobrush in all cows to determine mRNA expressions of melatonin receptor 1 (*MT1*), mouse double minute 2 (*MDM2*), MDM2 binding protein (*MTBP*), *BCL2-associated X*, apoptosis Regulator (*BAX*), p53 upregulated modulator of apoptosis (PUMA, gene symbol BBC3), mucin 1 (*MUC1*) and leukemia inhibitory factor (*LIF*). Plasma concentrations of melatonin were significantly greater in pregnant cows diagnosed pregnant at day 16 who sustained pregnancy to day 32 compared to nonpregnant cows at day 16, or pregnant at day 16 and who lost embryos by days 32. Concentrations of AOPP and TBARS were greater in nonpregnant cows at day 16 or pregnant at day 16 and who lost embryos by days 32 compared to those diagnosed pregnant at day 16 and who sustained pregnancy to day 32. In pregnant cows, endometrial mRNA expressions of *MDM2*, *MTBP*, *MTR1* and *LIF* were higher compared to pregnant–embryo-loss cows (*p* < 0.05). In contrast, mRNA expressions of *BBC3* and *MUC1* were greater at day 16 in pregnant–embryo-loss cows compared to pregnant cows (*p* < 0.05). In conclusion, melatonin status is a modulator of embryo well-being and maintenance of pregnancy in lactating dairy cows.

## 1. Introduction

Pregnancy is a critical stage of life, during which many physiological processes are modified. Although 85% of inseminations result in fertilization, less than 40% of cows calve after a first insemination [1]. The causes of embryonic loss and sustained pregnancy are multifaceted through maternal and embryonic factors. A considerable number of embryonic losses are attributed to maternal factors, such as an inability of the uterus to support conceptus growth and implantation [2,3,4] and increased oxidative stress [5]. Oxidative stress, inflammatory status, and mRNA expression of white blood cells in postpartum lactating cows are associated with reproductive responses after Timed AI [3].

Trophoblastic cells of the placenta produce melatonin and express *MT1* and *MT2* receptors [6]. Melatonin acts in an autocrine, intracrine and paracrine manner in the placenta [6,7]. Melatonin is known as a cell-protective molecule with different properties allowing it to exert strong antioxidant potential [8]. It is more effective in reducing free radicals than other well-known natural or chemically synthesized antioxidants [9,10,11]. Kivela et al. (1991) [12] demonstrated an increase in melatonin concentrations in the maternal blood during pregnancy, and Lanoix et al. (2008) [6] suggested that this increase is due to placental production of melatonin. Melatonin improves embryo quality through increasing expression of antioxidant genes that reduce mitochondrial damage and apoptosis [13].

Reactive oxygen species (ROS) may be responsible for causing increased embryo fragmentation, resulting from increased apoptosis [14]. The *TP53* gene codes for Tumor Protein P53 which works as a tumor suppressor and regulator of apoptotic processes [15]. The TP 53 protein acts transcriptionally to regulate expression of apoptotic proteins including *BAX***,** also known as bcl-2-like protein 4 (BAX), p53 upregulated Modulator of Apoptosis (*PUMA*; gene symbol, also known as Bcl-2-binding component 3 *BBC3*), E3 ubiquitin–protein ligase Mdm2 protein known as Anti-Apoptotic Mouse Double Minute 2 protein (MDM2, gene symbol *MDM2*), cell cycle proteins such as *p21* (product of the *CDKN1A* gene), and proteins associated with DNA repair such as the Growth Arrest and DNA-Damage-inducible 45 (*GADD45*) family [16,17]. Post-transcriptional actions of TP53, in which TP53 protein interacts directly with BAX and/or BCL2 proteins, also have been described. [18].

Cows with early embryonic loss were hypothesized to have both a higher oxidative status and lower concentrations of melatonin [6,12]. Melatonin is considered an essential protector of pregnancy that reduces oxidative stress, contributing to maintenance of pregnancy. This experiment investigated melatonin and its potential network of effects in intrauterine luminal cells that differ between lactating cows experiencing early embryonic loss compared to pregnant cows.

## 2. Material and Methods

### 2.1. Lactating Cows and Nutrition

This experiment was carried out during the summer at a commercial dairy farm of 3500 milking cows in the northern part of Iran (longitude and latitude, 36.33° N and 53.06° E). The Temperature Humidity index (THI) during the study was between 67 and 73. The photoperiodic conditions were 15 h light: 9 h dark (Sunrise: 6.00 a.m. and Sunset 9.00 p.m.). The study was conducted in accordance with the guidelines of the Iranian Council of Animal Care (1995) and approved by the ethics committee of Sari Agricultural Sciences and Natural Resources University (protocol #1998). All animal procedures were approved by the Iranian Ministry of Agriculture (Permission no. 2018.06.01).

Cyclic Holstein cows (*n* = 200, average milk production of 34.5 ± 0.6 kg/d and 3.1 ± 0.5 lactations) were enrolled in the study. All cows were evaluated and assigned a body condition score (BCS) on a scale of 1 to 5. An expert technician determined the BCS of cows. The mean BCS ± SEM of cows was 3.1 ± 0.1. Cows were fed a total mixed ration (TMR) formulated to meet the 2001 Nutrient Research Council (NRC 2001 [19] requirements for a lactating dairy cow of 700 kg body weight (BW) and producing 40 kg milk per day.

### 2.2. TAI Protocol

Ovaries were scanned at 30 ± 2 d postpartum using transrectal ultrasonography (Easi-Scan version 3, BCF Technology Ltd.; Livingston, Scotland, UK), and only cows that had a corpus luteum (CL) were enrolled in the study. All cows were synchronized by a MG6GP protocol: cows received PGF_2α_ (500 µg CLOPROSTENOL a synthetic analogue of prostaglandin F_2α_, Parnell Technologies, Australia), 4 days later GnRH (100 µg GONADORELIN ACETATE, Parnell Technologies), followed 6 days later by an Ovsynch56 TAI program, as described by Heidari et al. (2017) [20]. An experienced technician with commercially available frozen–thawed semen performed all inseminations.

### 2.3. Pregnancy Diagnosis

Pregnancy status at day 16 after TAI was predicted via blood cell *ISG15* mRNA gene expression. Prediction of pregnancy was based on blood cellular *ISG15* expression greater than −7.0 at day 16 after TAI [3,21]. Pregnancy at day 32 after TAI was diagnosed via transrectal ultrasonography. All cows were considered healthy (*n* = 200) and partitioned into nonpregnant at day 16 after TAI (*n* = 80), pregnant at day 16 (*n* = 120), pregnant at day 32 (*n* = 86), and cows experiencing embryonic losses between days 16–32 after TAI (*n* = 34)”.

### 2.4. Melatonin Concentrations

Blood samples were collected at 0, 16 and 32 days after timed (AI) (at 4.00 a.m., almost 2 h before sunrise) from all cows to determine melatonin concentrations. The enzyme-linked immunosorbent assay (ELISA) kit (IBL, Hamburg, Germany, CAT Number RE54021) was used to measure melatonin in plasma. IBL International-Tecan market a serum/plasma ELISA kit (RE54021) which uses a C18 reverse phase extraction procedure for the samples. The samples are applied to the columns and washed sequentially with water and 10% methanol, the melatonin eluted with methanol, the solvent evaporated, and the residue reconstituted for assay. The IBL melatonin antibody being used in the RIA and ELISA kits is reported to have a 1.2% cross reaction with N-acetyl serotonin and 2.5% for 5-methoxy tryptamine and 0.02% for serotonin, but presumably this is decreased following the extraction.

The resultant optical density was read at 405 nm (using 620 nm filter as a reference) on a Multiskan^®^ FC photometer (Thermo Fisher Scientific, Vantaa, Finland). The assay’s working principle is a basic competitive ELISA procedure in which the sample melatonin competes with a biotinylated melatonin for its antibody-binding sites (polyclonal; rabbit). The amount of biotinylated melatonin bound to the antibodies is inversely related to the amount of melatonin in the sample. Finally, the amount of melatonin in the sample is quantified by comparison with a standard melatonin dose–response curve (standard concentration 3.6, 10, 30, 100 and 300 pg/mL provided in the kit). Sensitivity of the melatonin assay was 1.6 pg/mL, and intra-assay and interassay coefficients of variation were 2–5% and 4–9%, respectively.

### 2.5. Measurements of Oxidative Status

Concentrations of AOPP (Advanced Oxidation Products of Proteins) were measured according to Witko-Sarsat et al. (1996) [22]. The intra-assay and interassay coefficients of variation were 1.02% and 2.27%, respectively. The AOPP values are expressed per unit volume (µmol/L), as well as per g of total protein (µmol/g). For this purpose, total protein concentration in plasma was determined with the Bradford method, (1976) [23] using BSA as standard. The intra- and inter-assay coefficients of variation were 1.42% and 4.16%, respectively. The TBARS (Thiobarbituric Acid Reactive Substances) test [24] was used for measuring oxidized lipids in plasma with a mean intra-assay CV of 7.01% and a mean interassay CV of 8.26%.

### 2.6. Endometrial Cells

Endometrial samples were collected from all cows at d 16 after AI using a Cytobrush Plus GT (Disposable cytology sampling brush 8”; Viamed Ltd., West Yorkshire, UK, [25]). Briefly, after caudal epidural anesthesia (induced with 5–10 mL lidocaine), the Cytobrush was passed through the cervix, and the tip of Cytobrush was placed in the uterine horn ipsilateral to the CL and against the antimetrial uterine wall. Endometrial cells were collected by rotating the Cytobrush three times clockwise while in contact with the uterine wall. Approximately 80% of cells sampled from the luminal endometrium are epithelial cells (Binelli M. and Co-workers personal communication; manuscript under review 2021). The Cytobrush is retrieved from the AI gun and the tip placed into a 2 mL cryo-tube containing 1 mL of trizol to release cells. The tube is frozen in liquid nitrogen for further storage at −80 °C.

#### 2.6.1. RNA Extraction and cDNA Synthesis

Total RNA was extracted from endometrial cells and used for cDNA synthesis using established protocols in our laboratory [26]. Briefly, RNA was isolated using a commercial kit (NucleoSpin RNA Blood, Cat No. 40200, Macherey-Nagel GmBH&Co. KG, Büren, Germany).

All RNA samples were quantified by spectrophotometry (#ND-1000, Nanodrop Technology Inc.; Wilmington, DE, USA), and the purification of RNA with A260/A280 ratio was between 1.8 and 2.0. Complementary DNA (cDNA) was synthesized from 150 ng RNA using a QuantiTect Reverse Transcription kit (Qiagen, Hilden, Germany, Cat No. 205314), as described by Dirandeh et al. (2021) [3].

#### 2.6.2. Real-Time PCR

Real-time PCR was performed using a 15 mL reaction volume containing 1 mL single-strand cDNA, 7.5 mL of 1 × SYBR Green master mix (Qiagen, GmbH, Germany, Cat. No. 204052), 1 mL of each forward and reverse primers and 4.5 mL of distilled H_2_O in a Rotor-Gene 6000 Real-Time PCR software (Corbett Research, Sydney, Australia) with specific bovine primers: melatonin receptor 1 *(MT1*, [27]), mouse double minute 2 (*MDM2*), MDM2 binding protein (*MTBP*), BCL2-associated X Apoptosis Regulator (*BAX*), p53 upregulated modulator of apoptosis (PUMA, gene symbol *BBC3*, [17]), Mucin 1 (*MUC1*, [28]) and leukemia inhibitory factor (*LIF*, [29]), in accordance with MIQE guidelines [30] and using following temperature program: 2 min at 50 °C, 10 min at 95 °C, 40 cycles of 15 s at 95 °C, and 1 min at 60 °C. The internal controls were *GAPDH*, *RPS9*, and *UXT* [3]. The geometric mean of the internal control genes was used to normalize the expression data. The relative levels of mRNA were analyzed by the 2^−ΔΔCt^ method [31].

### 2.7. Statistical Analysis

All data were analyzed using SAS (Windows; SAS Institute, Cary, NC, USA). Melatonin concentrations, total protein, AOPP and TBARS were analyzed using PROC MIXED of SAS (2001) with the following model:

Yijk = µ + αi + c(αj) + τk + ατ*ik + eijk

where µ is the population mean, αi is the pregnancy group effect, c(αj) is the cows (group) effect, which is the interaction for testing the pregnancy group effect, τk is the effect of sampling day after TAI, ατ*ik is the interaction effect of pregnancy group and sampling day after TAI, and eijk is the residual error for testing day and day * sampling effects. Pregnancy groups for the time analyses were nonpregnant (*n* = 114), pregnant (*n* = 80) and pregnant–embryo loss (*n* = 6).

Gene expression data for day 16 are presented as fold changes relative to one of the pregnancy groups. These were calculated using the method described by Yuan et al. (2006) [32].

Statistical analyses were performed on ΔCt values as described by Livak et al. (2001) [32]. Statistical differences were declared significant at *p* ≤ 0.05 and tendencies at *p* ≤ 0.10.

The simple correlations between mRNA expressions of uterine intraluminal cells and partial correlations adjusted for ISG15 expression of peripheral blood cells were analyzed using Pearson correlation analyses in SPSS 21 statistical software.

## 3. Results

Milk yields (kg/d) were similar between groups (pregnant = 45.3 ± 0.5, pregnant–embryo loss = 44.9 ± 0.7 and nonpregnant = 45.6 ± 0.8). Likewise, body condition scores were similar between groups (pregnant = 3.0 ± 0.25, pregnant–embryo loses = 3.25 ± 0.15 and nonpregnant = 2.9 ± 0.25).

**Figure 1:**
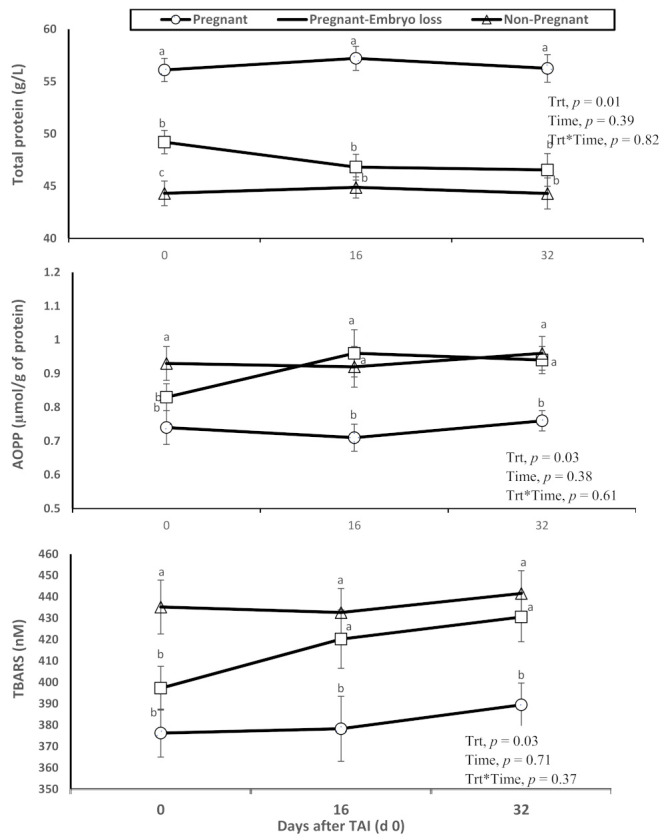
Changes in the systemic oxidative status (total protein content in plasma, advanced oxidation products of proteins (AOPP) and thiobarbituric acid reactive substances (TBARS)), of nonpregnant, pregnant and pregnant dairy cows with embryo loss between d 16 to 32 days after TAI (d 0). Values are means ± standard error of means. Different letters indicate a significant statistical difference in the specific day between the highlighted black symbols (*p* < 0.05).

The pregnant lactating dairy cows that had lower metabolic oxidation products (AOPP and TBARS) had higher overall total protein concentrations in plasma. Furthermore, relative differences were evident at TAI on day 0. Advanced oxidation protein products (AOPP) and TBARS (i.e., measurement of oxidized lipids due to lipid peroxidation) were greater in nonpregnant cows at day 16 or pregnant cows at day 16 who lost embryos by days 32, when compared to lactating cows diagnosed pregnant at day 16 who sustained pregnancy to day 32 (Figure 1).

**Figure 2:**
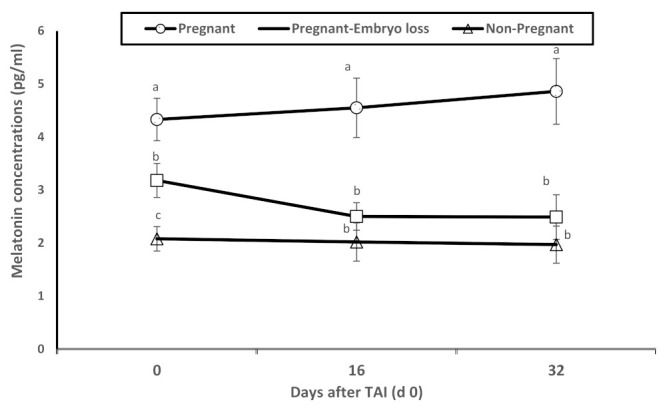
Mean plasma levels of melatonin (pg/mL) of nonpregnant, pregnant and pregnant dairy cows with embryo loss between d 16 to 32 days after TAI (d 0). Values are means ± standard error of means. Different letters indicate a significant statistical difference in the specific day between the highlighted black symbols (*p* < 0.05).

Dynamic changes in melatonin plasma concentrations were associated with pregnancy statuses. Melatonin concentrations were significantly greater in pregnant lactating cows diagnosed pregnant at day 16 with sustained pregnancy to day 32 compared to nonpregnant lactating cows at day 16 or pregnant at day 16 with lost embryos by days 32 (Figure 2). As observed in Figure 1, these differences were evident on day 0 at TAI.

**Figure 3:**
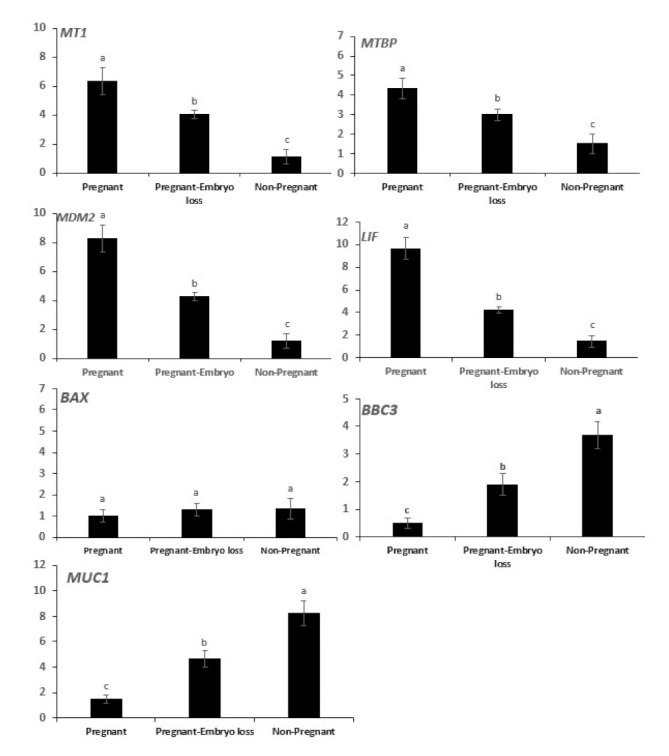
Relative mRNA expression of melatonin receptor 1 (*MT1*), mouse double minute 2 (*MDM2*), MDM2 binding protein (*MTBP*), DNA-damage-inducible 45 (*GADD45*), BCL2-associated X, apoptosis regulator (*BAX*), p53 upregulated modulator of apoptosis (*PUMA*, gene symbol *BBC3*), Mucin 1 (*MUC1*) and leukemia inhibitory factor (*LIF*) in day 16 endometrial samples of pregnant cows diagnosed pregnant at day 16 with sustained pregnancy to day 60 compared to nonpregnant cows at day 16 or cows pregnant at day 16 who lost embryos by days 32 or 60. Error bars represent the 95% CI of the adjusted fold change. Different letters indicate differences between groups (*p* < 0.05).

The mRNA expressions of *MT1*, *MTBP*, *MDM2*, *LIF* were greater in pregnant cows at day 16 and declined sequentially in pregnant cows that lost embryos, and they were lowest in nonpregnant cows at day 16 (Figure 3). Conversely, mRNA expressions of *BBC3* and *MUC1* were lowest in pregnant cows at day 16, they progressively increased in pregnant cows that lost embryos and were highest in nonpregnant cows. The expression of *BAX* mRNA was low and similar among the three groups (Figure 3).

Following the classification of pregnancy statuses, gene expression of *ISG15* in peripheral blood cells was used to distinguish pregnancy statuses of the three experimental groups. The array of gene expressions of intraluminal cells differed among the three pregnancy statuses (Figure 3). Associations of mRNA gene expressions among intraluminal endometrial cells and potential associated expressions with *ISG15* in blood cells among the 200 cows were evaluated. It was considered appropriate to report both simple (Table 1) and standard partial correlations between genes in luminal uterine endometrial cells adjusted for *IGF15* mRNA expression in blood cells (Table 2).

Among intraluminal endometrial cells, significant (*p* < 0.01) simple correlations (+ or -) were detected among all the genes (Table 1 Simple correlations). For example, positive correlations of *MDM2* expressions with *MTBP* (r = 0.982), LIF (r = 0.979) and *MT1* (r = 0.987) were detected (Table 1), as opposed to negative correlations of *MDM2* expressions with *BAX* (r = −0.574), *BBC3* (r = −0.983) and *Mucin* (r = −0.990). The cross section of gene associations with *BAX* were consistently lower. Of interest was that the *ISG15* gene expressions in peripheral blood cells at day 16 were correlated strongly with the full array of gene expressions of intraluminal endometrial cells (i.e., r = 0.89 to 0.973, or −0.972 to −0.977; Table 2). An examination of Standard Partial Correlations (Table 2) detected 6 of 21 correlations (29%) in which partial correlations adjusted for ISG15 (i.e., *MTBP/MUC1*, *MTBP/LIF*, *BAX/MUC1*, *BAX/LIF*, *BBC3/LIF*, *LIF/MT1*) were lower than the respective simple correlations (Table 1). This is indicative that *ISG15* gene expression in blood cells was associated with differential expression of specific genes in specific intraluminal endometrial cells.

**Figure 4:**
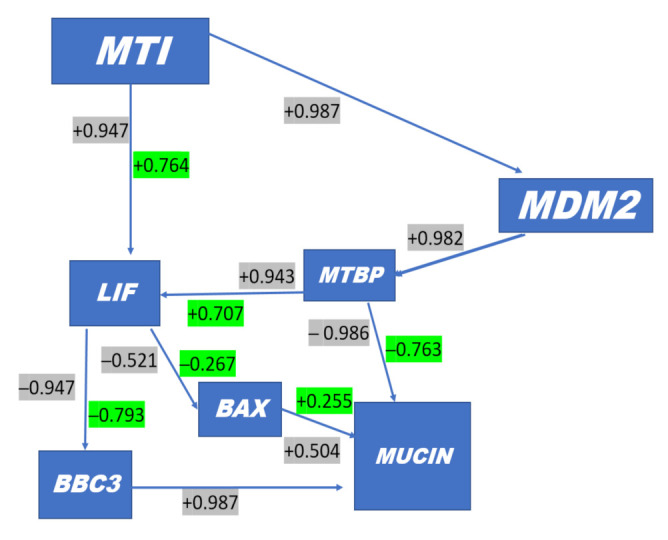
Simple (Gray) and Standard Partial correlations (Green) in uterine luminal endometrial cells responsive to ISG 15 expression in blood cells. Based on magnitude of decrease in correlation between simple and partial correlations, the systems in cellular target components are modulated by *ISG15* e.g., *MTBP* with *Mucin* is highly correlated negatively but when adjusted to average *ISG15* expression the relationship is reduced, which indicates that *ISG15* targets *MTBP*. In contrast, *MDM2* is highly negatively correlated with *MUC1*; however, when *MDM2* is negatively correlated with *MUC1* and adjusted for *ISG15*, the correlation is not reduced but it stays the same. + *MT1* Melatonin Receptor is found in peripheral tissues; + *MDM2* gene encodes the *MDM2* protein that binds and ubiquitinates p53 (i.e., inhibits *p53*) and stimulates degradation; + *MTBP* gene inhibits cell migration in vitro and suppresses the invasive behavior of tumor cells (by similarity). This may play a *role* in MDM2-dependent p53/TP53 homeostasis by binding of MDM2 to MTBP; -*BAX*, a proapoptotic activator, effects mitochondria membrane voltage-dependent anion channels (VDACs) involved in P54-mediated apoptosis; -*BBC3* gene encodes Bcl-2-binding component 3 protein; *BBC3* also known as *p53* upregulated modulator of apoptosis (*PUMA*); + *MUCIN* provides instructions for making a protein called mucin 1. This protein is one of several mucin proteins that make up mucus; + *LIF* gene encodes interleukin 6 family cytokine, involved in induction of hematopoietic differentiation, neuronal cell differentiation, and kidney development. This plays a role in immune tolerance at the maternal–fetal interface.

An overall proposed schematic representation depicting the direct protective and antiapoptotic pathways of melatonin within the bovine uterine endometrial cell is presented in Figure 4. The potential integrated associations of gene expression supporting the schematic are depicted in Figure 4. Increased plasma concentrations of melatonin at insemination and early pregnancy acting via increased *MT1* receptor expression enhances *MDM2* gene expression at day 16 (Figure 3). Gene expressions of *LIF* and *MDM2* appear to be central intracellular focal points leading to an ultimate decrease in gene expression of mucin. This appears to be mediated by a *MDM2*-induced increase in *MTBP* expression acting in concert with *ISG15* expression leading to a decrease in Mucin expression. Concurrently, increased *MT1* expression acting in concert with *ISG15* expression enhances expression of *LIF* that targeted a moderate decrease in *BAX* gene expression. The decrease in *BAX* expression would reduce the positive effect of *BAX* on Mucin expression. An additional critical factor intracellularly is the degree of mitochondrial regulation of apoptosis. High expression of *LIF* in pregnancy at day 16 is associated with low gene expression of *BBC3* in the two nonpregnant statuses (Figure 3). Expression of *BBC3* is a major upstream regulator of the apoptotic pathway in mitochondria that ultimately influences both nuclear and mitochondrial pathways leading to cellular apoptosis. The high *LIF* expression associated with a negative decrease in *BBC3* expression (Figure 3 and Figure 4) exerts an antiapoptotic effect, partly associated with ISG15 expression in blood cells.

## 4. Discussion

Oxidative stress (OS) is due to an imbalance between oxidants and antioxidants [33]. Due to abrupt and sustained environmental changes (e.g., THI), at the time of insemination and pregnancy, etc., transitioning from hypoxic to hyperoxic status increases reactive oxygen species (ROS) of the maternal–conceptus cells. This transition causes OS and atrophy of cells resulting in death of the localized conceptus (embryo and endometrial interface) [34].

The relatively greater values of TBARS and AOPP in cows with early embryonic loss are indicative of oxidative damage of lipids (TBARS) and proteins (AOPP) (Figure 1). Similar results were described by Dirandeh et al. (2021) [3] reporting decreased total antioxidant capacity (TAC), SOD and GPx in cows with embryonic loss, as well as increased lipid peroxidation. Zhao et al. (2019) [35] reported that GPx abundance was greater in cyclic cows compared to cows with inactive ovaries, suggesting that OS in ovaries may lead to failure in follicular development to form a cumulus structure that promotes oocyte production.

**Figure 5:**
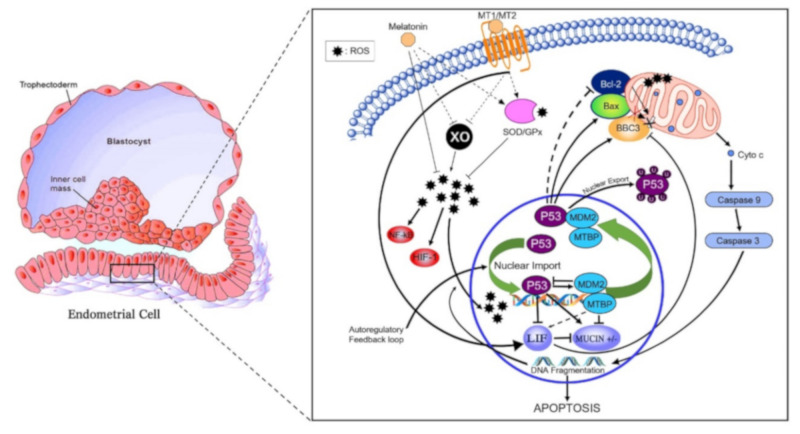
Proposed antiapoptotic pathway of melatonin in the bovine endometrial cell. Abrupt environmental change at pregnancy from hypoxic to hyperoxic induces xanthine oxidase (XO) expression and activity, thereby generating reactive oxygen species (ROS). ROS-induced DNA fragmentation also activates p53. Regulation of the Bax/Bcl-2 ratio pathway leads to the activation of caspase 9 and caspase 3. Downstream effects of caspase 3 include increasing DNA fragmentation. Melatonin, via its receptors, indirectly increases expression and activity of superoxide dismutase (SOD) 1 and 2 as well as glutathione peroxidase (GPx) antioxidant enzymes. XO expression and activity is indirectly reduced by melatonin and its receptors. Moreover, melatonin signaling creates a positive feedback loop between MDM2/MTBP that regulates p53 by two main mechanisms: inducing p53 ubiquitination that suppresses the transactivation domain of p53, hence preventing transcription of downstream targets (i.e., increase Bcl-2: Bas ratio and BBC3); secondly, melatonin binding to MT1 increases LIF. The increase in LIF decreases BBC3 therefore reducing apoptosis. The decrease in BBC3 expression reduces the positive regulation of MUC1 expression thus allowing cell adhesion.

In the present study, plasma concentrations of melatonin were significantly greater in pregnant cows compared to nonpregnant cows at day 16 or cows pregnant at day 16 with lost embryos by day 32 (Figure 2). Melatonin, through direct scavenging and indirect antioxidant actions, limits oxidative stress in all cells and protects DNA and other components from damage [36]. The antioxidative actions of melatonin and its metabolites are extremely vast, including ability to neutralize ROS [10]. Melatonin also directly and indirectly increases antioxidant enzyme expressions of superoxide dismutase (SOD) and glutathione peroxidase (GPx) (Figure 5). These are bifunctional effects of directly increasing enzyme expressions associated with detoxification of ROS and increasing mRNA expressions of antiapoptotic pathways of melatonin via *MT1* and *MT2* (Figure 5). Use of melatonin in embryo culture has caused reductions in oxidative stress and apoptosis [37]. Moreover, melatonin has increased the number of blastocyst cells, glutathione activity, and reduced both oxidative stress and apoptosis [38]. In rats exposed to oxidative stress induced by sodium fluoride (NaF), melatonin improved levels of catalase (CAT) and superoxide dismutase (SOD), which counteracted ROS activity on embryos [13].

In the present study, mRNA expression of *MTI* receptor in uterine luminal epithelial cells was significantly greater in pregnant cows compared to nonpregnant cows at day 16 or cows pregnant at day 16 but who lost embryos by days 32 (Figure 3). In addition to the direct antioxidant activity and inhibition of xanthine oxidase (XO), melatonin-induced functions also are mediated through binding to melatonin membrane receptors MT1 (Figure 5) and MT2 [11]. Following melatonin stimulation, MT1 and MT2 receptors regulating antioxidative and antiapoptotic signaling pathways involved in embryo implantation were identified, e.g., expressions of *p53*, *MUC-1* and *LIF* (Figure 5). It has been shown that differential expression of *MT1* and *MT2* receptors in pregnant and nonpregnant human uteri are able to influence the cyclic rhythm of myoendometrial contractility [39]. This is an insightful response because it infers not only melatonin action within the control systems of the brain, but direct melatonin regulation of tissues such as the uterus. Serotonin, the precursor to melatonin, has a dual control system based on differential expression of two isoforms of tryptophan hydroxylase (TPH1 and TPH2) [40]. Indeed, TPH1 is highly expressed throughout a plethora of tissues involved in regulation of metabolism (e.g., small intestine, stomach, ovary, uterus, etc.), However, TPH2 expression was much more restricted to tissues of the central nervous system. Melatonin, as a product of serotonin biosynthesis, has a major metabolic role in the body, and its biosynthesis is not restricted to the pineal but is produced profusely in a diversity of tissues. This is evident by differences detected in plasma melatonin concentrations in highly productive fertile cows compared to cows that did not readily conceive or had embryonic mortality (Figure 1). The high-producing dairy cow is challenged with the metabolic demands and managing oxidative stress. Zhao et al. (2019) [35] characterized an array of differential blood proteins in dairy cows with inactive versus active cyclic ovaries during early lactation and reported that GPx was downregulated in cows with inactive ovaries. Consequently, OS results in lower quality of subsequent ovulatory follicles and consequently lower embryo quality. Distinct differences in antioxidative statuses and differential gene expressions of cells harvested from the luminal endometrial environments at day 16 were detected when exposed to the conceptus compared to nonpregnant cows or pregnant cows losing embryos (Figure 3). Studies performed in a mouse culture system displayed that melatonin stimulates formation of blastocysts during embryogenesis [41]. From a molecular perspective, melatonin regulates functionality or activation of p53 by inducing p38-dependent phosphorylation of p53 [42]. Damaged DNA leads to activation of p53 phosphorylation, which increases the ability of p53 to bind DNA and mediate transcriptional activation. This p53-dependent DNA-damage response to activate transcription is mediated by MT1 and MT2 in mice [43]. The upregulation of p21 increased p38 activation that favors p53 phosphorylation and increased transcriptional activation [44]. These results indicate that p53 phosphorylation could be a downstream element responsive to MT1–MT2 receptor binding to melatonin. Transcription of BBC3 is increased when exposed to diverse apoptotic stimuli (i.e., DNA-damaging agents) and wild-type p53 induces apoptosis via the mitochondrial pathway. Indeed, *BBC3* expression was low in endometrial cells of pregnant cows and increased sequentially in pregnant–embryo-loss and nonpregnant cows. BCL2 prevents BAX/BAK oligomerization, and *BAX* gene expression was low in all three groups. Furthermore, BCL2 binds to and inactivates BAX as well as other proapoptotic proteins to inhibit apoptosis. These coordinated dynamics would contribute to the differential responses among pregnancy statuses.

LIF expression promotes cell survival by suppressing apoptosis and positively influences embryo implantation [45]. This is supported by parallel increases in *MT1*, *MTBP*, *MDM2* and *LIF* gene expressions in luminal epithelial cells from pregnant cows at day 16 compared to nonpregnant cows (Figure 3).

Apoptosis can occur through several pathways, and the present study identified the integrated *MDM2-BBC3* and LIF expression pathways as a likely target of melatonin action in bovine endometrial cells. In pregnant cows, increased plasma melatonin and endometrial *MT1* expression were associated with increased expressions of *MDM2*, *MTBP* and *LIF,* that directly and indirectly mediated downstream decreases in expression of both apoptotic *BBC3* and *BAX* genes. LIF-induced reductions in gene expression of both *BBC3* and *BAX* are considered important, since each would have ultimate net positive associations to reduced *MUC1* expression. In contrast, the upstream *MTBP* gene may directly target a decrease in *MUC1* expression. Net reduction in mucin expression is considered essential for adhesion of trophoblast cells to underlying endometrial cells. Consequently, these associated pathways ultimately target a decrease in mucin expression in pregnancy versus increases of expression in cows losing embryos or nonpregnant cows. Furthermore, increases in ISG15 of blood cells significantly enhanced these negative associations as well. Day 16 is the time associated with the start of the process of pregnancy recognition in dairy cows. Other luminal endometrial epithelial cells throughout the uterine horn not adjacent to the trophoblast cells would remain coated with mucin [46]. Cells harvested locally within the uterine horn of projected pregnancy provided a population of cells differentially expressing a complement of antioxidant/antiapoptotic genes associated with pregnancy statuses (pregnant, pregnant–lost embryos and nonpregnant), and expression of these genes were associated with *ISG15* expression + and ─in peripheral blood cells.

MDM2 protein interacts with several intracellular proteins, one of which is MTBP that is expressed highly in the ovary [47]. MTBP inhibits growth and induces G1 arrest in a TP53-independent manner in human lung carcinoma and osteosarcoma cell lines [46], and promotes MDM2 degradation of TP53 protein [48]. The increase in *MTBP* mRNA expression in pregnant cows in the present study may ultimately potentiate the inhibitory effect of melatonin on MDM2-dependent apoptosis. Furthermore, increased expression of *MTBP* appears to decrease gene expression of *MUC1*. We are not aware of any other reports suggesting regulation of *MTBP* by melatonin in the uterus. Perhaps regulation of the *MTBP* mRNA expression and interaction with *MDM2* are related to cell/tissue remodeling dynamics of the developing endometrium/embryo/conceptus in early pregnancy (e.g., >d16), and this warrants further investigation.

In contrast to the potential stimulatory effects of melatonin on increased expression of *MDM2* and *MTBP* mRNA in pregnant cows (Figure 3), expression of mRNA encoding the potent proapoptotic protein *BBC3* was inhibited by P53 in pregnant cows, as shown Figure 3 and Figure 4. Consequently, expression of *BBC3* increased progressively in pregnant cows that lost embryos to even higher expression levels in nonpregnant cows (Figure 3). This protein is the major mediator of TP53-induced apoptosis, and ultimately activates BAX protein at the mitochondrial membrane [49]. However, *BAX* gene expression in all three reproductive statuses was low (Figure 3). Expression of *BAX* was weakly associated with *BBC3* gene expression in the endometrium (Table 1) and was not altered by *ISG15* expression in blood cells. (Table 1 and Table 2).

In present study, mRNA expression of *LIF* was greater, whereas *MUC1* expression was lower at day 16 in pregnant cows, as shown in Figure 3. One of the main estrogen mediators responsible for implantation seems to be leukemia inhibitory factor (LIF), regulated by p53 [50], that supports uterine receptivity during implantation. However, plasma estradiol and estrone concentrations are low in early pregnant cows [51]. Carlomagno et al. (2018) [45] showed implant failure in mice when LIF was suppressed. Mucin (MUC1) is also a regulatory molecule, which is expressed and secreted from endometrial cells to inhibit cell–cell adhesion [52]. Expression of *MUC1* in the endometrium has been suggested to create a barrier to embryo adhesion that must be suppressed during implantation [53]. Interestingly, when human blastocysts were allowed to attach to the endometrial epithelial cell monolayer, MUC1 was removed locally at the implantation site. Pregnancy recognition in cattle is associated with adherence and coregulation between trophoblast and endometrial cells [54]. For example, a plethora of endometrial adhesion genes were expressed less in pregnant cows compared to cyclic cows at day 16, see Supplementary file [54]. This would support the idea that cell-to-cell adherence in nonpregnant or pregnant cows that lost embryos in early pregnancy may be partially due to copious gene expression of adhesion molecules. Likewise, a complement of three mucin genes were upregulated and three were downregulated for expression of mucins [54]. Differential expression of these mucin genes co-expressed at the interface of the luminal endometrium with the trophoblast compared to luminal epithelial cells nonadjacent to the trophoblast would be insightful. Indeed, associations of endometrial epithelial gene expressions within apoptotic pathways regulated by melatonin were modulated in association with *ISG15* concentrations in blood cells (Table 1 and Table 2; Figure 3). These associations with *ISG15* in endometrial cells are due to interferon T secretion by day 16 of pregnancy. This is further substantiated by measurements of ISG15 in blood cells as a predictor of pregnancy status in the present experiment with 200 cows.

Kasimanickam and Kasimanickam, (2021) [55] reported that *MUC1* was lower in embryos of grade 1 (excellent) and 2 (fair) compared to 3 (poor) and 4 (unfertilized/dead/degenerate). Similarly, expression of *MUC1* was lower in endometrium of cows without subclinical endometritis compared with those that had subclinical endometritis. Kasimanickam et al. (2014) [28] reported greater expression of *MUC1* in cows with metritis and endometritis compared to normal cows, which subsequently resulted in poor reproductive performance, possibly due to embryonic death.

The present findings indicate that proper balance and control of OS and ROS are essential for health and productivity of the lactating dairy cow, but there is also a local regulated antioxidative system within the maternal–conceptus unit at the time of pregnancy maintenance or loss in early pregnancy. A major consideration for reproductive management of lactating dairy cows is management of oxidative stress through regulation of melatonin synthesis and secretion, which directly influences antioxidant activity for optimal conceptus development. This system appears to regulate overall metabolism and health under dual control of central nervous system and peripheral tissue metabolism. This is exemplified by the sequential homeorhetic/homeostatic processes occurring postpartum, to ultimately permit a uterine–conceptus dialogue for a successful pregnancy concurrently with meeting the needs of the lactating dairy cow. Further noninvasive strategies of melatonin regulation entail regulation of photoperiod and dietary nutritional supplementation of amino acids (i.e., differential tissue conversion of tryptophan for synthesis of serotonin, the immediate precursor of melatonin) that warrant further investigation. Such strategies may complement and/or advance current antioxidant strategies mediated through vitamin (s) and supplemental nutraceutical inputs, such as feeding organic selenium-methionine and bypassing fatty acid supplements that increase availability of n-3 fatty acids to improve antioxidant status.

An overarching finding of this experiment is the potential roles of ISG15 secreted locally by the endometrium in apposition to trophoblast cells of the elongating embryo at day 16. This dialogue is essential for maintenance of pregnancy and distal effects such as lifespan of the corpus luteum. A role for ISG15 in regulating oxidative statuses of the maternal–conceptus unit is documented, linking ISG15 gene expression in blood cells with antioxidant and antiapoptotic regulatory genes of uterine luminal epithelial cells recovered with Cytobrush sampling within the potential 16-day-pregnant uterine horn.

The process in which ISG15 catalyzes post-translational conjugation to de novo synthesized intracellular proteins is termed ISGylation, which regulates stability and functional processes [56,57,58,59]. Consequently, a functional role of ISG15 may involve mitochondria, where a plethora of 52-ISGylated proteins were predicted to localize and impact various intracellular systems such as negative regulation of apoptotic signaling pathway, ATP biosynthetic processes, oxidation-reduction status, TCA cycle and glycolysis.

These novel findings emphasize the potential importance of ISG15 in the control of cellular metabolism and mitochondrial responses such as oxidation status and immune responses. A better understanding of how regulation of ISG15 affects downstream oxidation status, immune functions, reproductive processes and their interactions is needed. An overall melatonin-regulatory system may have a profound effect on physiological statuses associated with lactation, environmental heat stress, and nutritional management, genomic differences acquired from genetic selection, production medicine/management programs and reproductive performance. This is particularly important with ongoing global climatic change. In the lactating dairy cow, a localized operational control system involving melatonin appears to be functional in the uterine–ovarian housing system for protection of the developing conceptus and its maintenance during early pregnancy. This system is most likely sustained through the transition period, encompassing parturition and recrudescence of postpartum fertility leading to a pregnancy.

## 5. Conclusions

During the preimplantation period, oxidative stress negatively influences embryo quality and pregnancy success in lactating dairy cows. At day 16 of pregnancy, peripheral plasma markers of antioxidant status and concentrations of melatonin and localized gene expression of antioxidant cellular markers in uterine luminal cells were associated with subsequent fertility responses. Gene expression responses of uterine luminal cells were associated with gene expression of ISG15 in blood cells. Beneficial responses of melatonin status towards fertility support potential nutraceutical and other strategies to enhance availability and biosynthesis of melatonin, with the goal of enhancing reproductive performance of high producing dairy cows.

## Figures and Tables

**Table 1:** Simple correlations of genes expressed in uterine luminal cells collected at day 16 after insemination from pregnant, pregnant with embryo loss, and nonpregnant lactating dairy cows.

	*MDM2*	*MTBP*	*BAX*	*BBC3*	*MUC1*	*LIF*	*MT1*
*MDM2*	1						
*MTBP*	0.982 **	1					
*BAX*	−0.574 **	−0.604 **	1				
*BBC3*	−0.983 **	−0.995 **	0.566 **	1			
*MUC1*	−0.990 **	−0.986 **	0.504 **	0.992 **	1		
*LIF*	0.979 **	0.943 **	−0.521 **	−0.947 **	−0.965 **	1	
*MT1*	0.987 **	0.992 **	−0.566 **	−0.993 **	−0.993 **	0.947 **	1
*ISG15*	0.939 **	0.969 **	−0.464 **	−0.977 **	−0.972 **	0.891 **	0.973 **

Melatonin receptor 1 (*MT1*), mouse double minute 2 (*MDM2*), MDM2 binding protein (*MTBP*), *BCL2-associated X*, apoptosis regulator (*BAX*), p53 upregulated modulator of apoptosis (PUMA, gene symbol BBC3), Mucin 1 (*MUC1*) and leukemia inhibitory factor (*LIF*). ** represents a (*p* < 0.01) degree of significance for the correlations between the 2 variables identified.

**Table 2:** Standard partial correlations ^1^ of genes expressed in uterine luminal cells collected at day 16 after insemination from pregnant, pregnant with embryo loss, and nonpregnant lactating dairy cows.

	*MDM2*	*MTBP*	*BAX*	*BBC3*	*MUC1*	*LIF*	*MT1*
*MDM2*	1						
*MTBP*	0.853 **	1					
*BAX*	−0.456 **	−0.709 **	1				
*BBC3*	−0.893 **	−0.912 **	0.603 **	1			
*MUC1*	−0.956 **	−0.763 **	0.255 ^n.s^	0.840 **	1		
*LIF*	0.914 **	0.707 **	−0.267 ^n.s^	−0.793 **	−0.926 **	1	
*MT1*	0.930 **	0.863 **	−0.560 **	−0.856 **	−0.864 **	0.764 **	1

^1 ^If the partial correlation, r_12.3_, is smaller than the simple (two-variable) correlation r_12_, but greater or less than 0, then variable 3 (i.e., ISG15) partly explains the correlation between X and Y. This co-responsiveness with ISG15 occurred in 38% of the correlation pairs, i.e., 6/21 = 29%, identified by Grey Boxes. Melatonin receptor 1 (*MT1*), mouse double minute 2 (*MDM2*), MDM2 binding protein (*MTBP*), *BCL2-associated X*, apoptosis regulator (*BAX*), p53 upregulated modulator of apoptosis (PUMA, gene symbol BBC3), Mucin 1 (*MUC1*) and leukemia inhibitory factor (*LIF*). ** represents a (*p* < 0.01) degree of significance for the correlations between the 2 variables identified.

## Data Availability

The data that support the findings and which are presented in this study are available on request from the corresponding author, Essa Dirandeh, dirandeh@gmail.com. The data is not publicly available as not all data of the study have been published yet.
